# Characterization and phylogenetic analysis of the complete mitochondrial genome of *Prolixicheilus longisulcus* (Teleost: Cyprinidae)

**DOI:** 10.1080/23802359.2020.1788448

**Published:** 2020-07-11

**Authors:** Lei Zhou, Shihui Huang, Tianxu Kuang, Yusen Li

**Affiliations:** a Joint Laboratory of Guangdong Province and Hong Kong Region on Marine Bioresource Conservation and Exploitation, College of Marine Sciences, South China Agricultural University, Guangzhou, China; b Guangdong Laboratory for Lingnan Modern Agriculture, Guangzhou, China; c Guangxi Key Laboratory of Aquatic Genetic Breeding and Healthy Aquaculture, Guangxi Academy of Fishery Sciences, Nanning, China

**Keywords:** *Prolixicheilus longisulcus*, mitochondrial genome, phylogenetic analysis

## Abstract

In this study, we assembled the complete mitochondrial genome of *Prolixicheilus longisulcus* based on high-throughput Illumina sequencing. The complete mitochondrial genome of *P. longisulcus* was 16,598 bp in size. It was made up of 13 protein-coding genes, 22 tRNA genes, 2 rRNA genes, and 1 control region. The overall base composition included A(32.38%), T(26.08%), C(26.32%) and G(15.22%). The phylogenetic tree was conducted to provide a relationship within Labeoninae based on the maximum likelihood (ML) method. The ML tree demonstrated this species had the closest relationship with *Cophecheilus bamen*. These results should help to better understand the phylogenetic evolution of Labeoninae.

*Prolixicheilus longisulcus* is an endemic species living in the karst regions of South China. It was first found in Guangxi, China, in August 2008 and was considered as a new species of the genus *Pseudogyrinocheilus* for its special oromandibular morphology shared with *Pseudogyrinocheilus prochilus* (Zheng et al. 2010). However, later molecular results revealed two distant lineages between these two species (Zheng et al. 2016). Therefore, *Prolixicheilus longisulcus* was erected for *Pseudogyrinocheilus longisulcus.* In this study, we sequenced the mitochondrial genome of *P. longisulcus* to provide insight into the phylogenetics of Labeoninae that is essential for taxonomic, systematic, and population genetic studies.

The specimen was collected from a tributary of Pearl River in Jingxi City, Guangxi Province, China (E106.298, N23.137) in September 2019 and maintained in South China Agricultural University (accession no. SCAU-20190912001). The complete mitogenome was sequenced by next-generation sequencing using the Illumina HiSeq2500 instrument (Illumina, Inc., San Diego, CA, USA) with the de novo assembly strategy (Yang et al. 2018).

The mitochondrial genome of *P. longisulcus* was 16,598 bp (GenBank Accession number: MT157615) in length, with an A + T content of 58.46% and its base composition: 32.38% A, 26.08% T, 15.22% G, and 26.32% C. Like other vertebrates (Boore 1999), it has 13 protein-coding genes (PCGs), 22 tRNAs, 2 rRNAs, and 1 putative control region (CR). Among all the PCGs, there were 12 (nad2, cox1, cox2, atp8, atp6, cox3, nad3, nad4I, nad4, nad5, cob, and nad1) on the heavy strand, while one (nad6) on the light strand. The PCGs used two different start codons, i.e. ATG and GTG. All of them ended with TAA, TA–, or T–– stop codon.

We found that the 22 Labeoninae species can be divided into two branches with *Oreochromis niloticus* as the outer group (Figure 1). All the species of *Labeo* formed branch one. The other 10 genera clustered into branch two. Phylogenetically, *P. longisulcus* and *Cophecheilus bamen* were the closest relatives with 99% support value.

**Figure 1.**
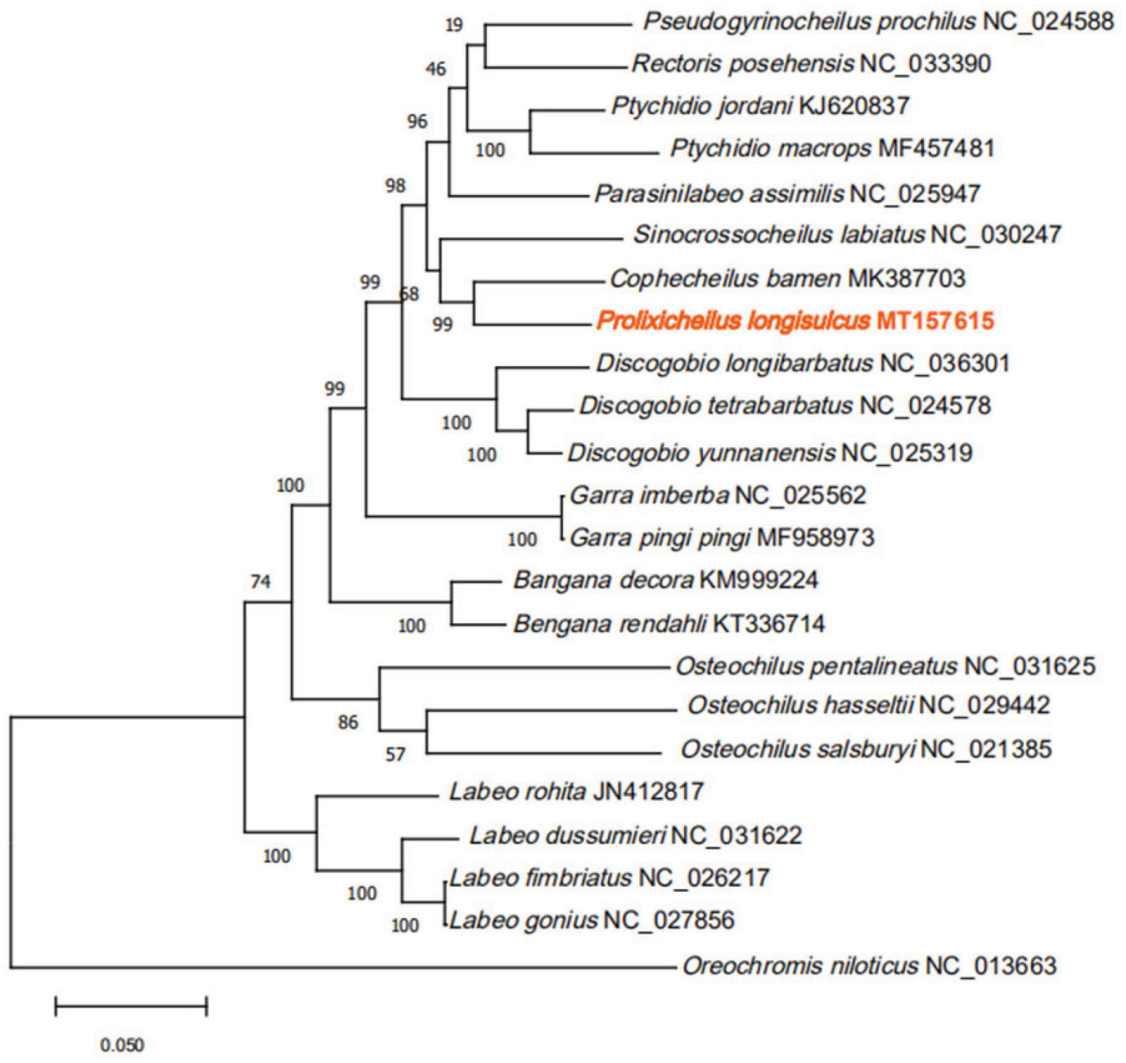
Phylogeny of 22 species within the subfamily Labeoninae based on the maximum likelihood analysis of 13 mitochondrial protein-coding genes. The support values are shown next to the nodes. *Oreochromis niloticus* (GenBank: NC_013663) was included as the outgroup taxon.

In conclusion, we sequenced, annotated, and characterized the complete mitogenome of *P. longisulcus*. This research should provide valuable information for exploring the genetic diversity and phylogeny of the Labeoninae subfamily.

## Data Availability

The data that support the findings of this study are openly available in GenBank of NCBI at https://www.ncbi.nlm.nih.gov, reference number MT157615.
